# Covid-19 in Children and Young Adolescents in Al Ain, United Arab Emirates- a Retrospective Cross-Sectional Study

**DOI:** 10.3389/fped.2020.603741

**Published:** 2021-01-18

**Authors:** Ahmed Elghoudi, Huda Aldhanhani, Ghassan Ghatasheh, Elsadeq Sharif, Hassib Narchi

**Affiliations:** ^1^Department of Pediatrics, Al Ain Hospital, Al Ain, United Arab Emirates; ^2^The Department of Paediatrics, College of Medicine and Health Sciences, UAE University, Al Ain, United Arab Emirates; ^3^Department of Pediatric, Sheikh Khalifa Medical City, Abu Dhabi, United Arab Emirates; ^4^Paediatric Infectious Disease Unit, Child Health Institute, Tawam Hospital, Al Ain, United Arab Emirates

**Keywords:** 2019-nCoV, children, coronavirus, COVID-19, SARS-CoV2, prevalence

## Abstract

**Aim:** In this retrospective single-center study, we report our experience with a cohort of children admitted to our hospital in Al Ain City, United Arab Emirates, with confirmed COVID-19. We also compare our findings to similar reports in the literature.

**Patients and Methods:** Between 1st March and 31st May 2020, we reviewed the electronic patient medical records of all children with confirmed COVID-19 (ICD-10 code U07.1) managed in Al Ain hospital, designated as the only COVID-19 center in the city.

**Results:** There were 288 children admitted with a confirmed diagnosis of Covid-19 during the study period (mean age 7.3 years, median 6.5, range 1 month to 16.9 years). The age-specific point prevalence was the highest under the age of 5 years (mean 2.0 per 1,000, 95% ci 1.7, 2.4) and decreased progressively to 0.6 per 1,000 (95% ci 0.4, 0.9) over the age of 14 years. Hospital admission was required for 193 (67%) children while 95 (33%) were discharged from the emergency department. Most children (*n* = 214, 74%) had been exposed to a family member with suspected or confirmed COVID-19 and asthma which was the most frequent comorbidity (*n* = 37, 13%). The most common symptoms were cough (*n* = 130, 45%), fever (*n* = 14, 4.8%), upper respiratory tract infection (*n* = 93, 32.3%), and lower respiratory tract infection in 28 (9.7%). None of the children presented with acute respiratory distress syndrome, neurological symptoms, sepsis, or septic shock. Neutropenia (absolute neutrophil count or ANC< 1.5 × 10^9^/L) was observed in 10.4% and thrombocytopenia (<150 platelets × 10^9^/L) in 72% of children. Nineteen patients (9%) had abnormal imaging studies (chest X-ray and chest computed tomography). Abnormalities were bilateral in six (43%), right-sided in seven (50%) with only one child (7%) with left-sided involvement. None of the children required invasive respiratory support, but four (1.4%) required noninvasive respiratory support. The median length of hospital stay was 3.3 days [1.9, 5.9]. There were no deaths in the hospital even in those with comorbidities.

**Conclusions:** Our results confirm previous reports of mild illness of COVID-19 in our child population, even in those with comorbidities. The age-standardized prevalence was higher in children (<5 years) compared to young adolescents.

## Introduction

Since the first case report of the 2019 novel coronavirus disease (COVID-19) in December 2019 in Hubei, Wuhan, China, the outbreak has now spread to most countries worldwide (1) including the United Arab Emirates (UAE) where the first confirmed case was announced on 29th January 2020. Although its agent, the Severe Acute Respiratory Syndrome Coronavirus 2 (SARS-CoV-2), has affected all ages, it became apparent that most children remained asymptomatic while a few developed mild disease in contrast to severe illness in older people and those with comorbidities (2). In symptomatic children, the disease manifests as an influenza-like illness with mild upper respiratory tract infection (URTI). In the few with more severe illness, pneumonia is the most common complication, especially in those with comorbidities.

The reasons for a milder illness in children compared to older adults remain largely unknown. One possible explanation was attributed to the lower expression of the angiotensin-converting enzyme (ACE) 2 receptors in children compared to adults (3). These ACE 2 receptors are required by the virus to adhere to the cells in the respiratory tract and produce infection (4). Another hypothesis is that children demonstrate a qualitatively different immune response compared to adults (5). In addition, it has been demonstrated that many other viruses commonly colonize the children's airway mucosa, thus competing and interfering with SARS-CoV2 reproduction and spread in their respiratory tract cells (6). The consensus seems, therefore, to be a combination of differences in the response, function, and composition of the immature immune system of children (7).

The predominance of asymptomatic or mildly symptomatic children with COVID-19 should not be a reason for complacency, because of the silent risk of transmission to adults and elderly which could potentially result in the continuous spread of the disease (8).

Previous reports from many countries have been published on children with COVID-19, with most being case series with small-sized samples, except from China (9–17). There have been, so far, no data published from the UAE, a unique multi-ethnic society with a high prevalence of multigenerational extended families living at very close proximity to each other. With the constant worry about a prolonged pandemic and possible subsequent waves of infection, every healthcare system needs to plan how to manage future outbreaks. This requires, in the first instance, quantification of the burden of COVID-19 in their society, establishment of age-specific prevalence, and assessment of the prevalence of complications in order to plan for the resources required for adequate management.

Al Ain Hospital (AAH) is a teaching general hospital in the city of Al Ain serving a population of nearly 500,000. On 23rd March 2020, AAH became the sole designated facility in the city of Al Ain for COVID-19 patients of all ages. All non-COVID-19 patients were then transferred out of AHH to other health facilities to enable non-COVID-19 services, including elective and urgent care, to take place within a safe environment and avoid cross infection with the highly infectious SARS-Cov2 virus. All confirmed/suspected cases of COVID-19, presenting either to any public or private health facility in the city throughout that period, were transferred to AAH. The initial bed capacity of 324 was also increased to 415 beds, including 85 negative-pressure intensive care beds with 54 beds designated for children tested positive to SARS-Cov2. This gave us a unique opportunity to describe the prevalence of COVID-19 in the pediatric population in Al Ain city in the UAE and who were managed in the single designated referral hospital for COVID-19 in the city. We describe their clinical presentation, laboratory features, complications observed, and outcomes, comparing them to previous reports in the literature.

## Materials and Methods

### Design and Settings

This is an observational retrospective cohort study of children under 16 years of age who were managed at Al Ain hospital (AAH), with suspected or confirmed COVID-19, between 1st March and 31st May 2020, the period when the pandemic peaked in the UAE, similar to many other countries. The study included all those managed in the emergency department as well as those admitted to the hospital.

Some children were brought by their parents to the emergency department because of febrile illness and/or sore throat, suspected as symptoms of COVID-19, while other asymptomatic children were brought for assessment following contact with a confirmed case. The inpatient wards also received direct referrals from other hospitals, local ambulatory health service centers, and COVID-19 screening hubs stationed throughout the city.

Medical care provided to the patients remained exclusively at the discretion of the attending physician, and no interventions were administered solely for the purpose of this study.

### Ethical Approval

This was granted by the Abu Dhabi COVID-19 Research Institutional Review Board and informed consent was waived as it was a retrospective study with all collected data being anonymized.

### Procedure for SARS-Cov2 Diagnosis

The diagnosis of SARS-Cov2 was established as per the World Health Organization (WHO) and the local health authorities' guidance, using nasopharyngeal real-time polymerase chain reaction (RT PCR) (Supplement 1).

### Data Management

All children diagnosed with COVID-19 during the study period were enrolled in the study by searching the hospital electronic medical records for ICD10 code U07.1. The medical records of the affected children were reviewed to document their age, nationality, history of contact or exposure to COVID-19 case, symptomatology, comorbidities, vital signs on presentation, and the results of the laboratory tests mandated by the hospital for investigating patients with COVID-19. These investigations included complete blood count (CBC), serum levels of urea, creatinine, liver enzymes, ferritin, and lactic dehydrogenase (LDH), as well as coagulation screen, glucose-6-phosphate dehydrogenase level, and chest x-ray. We also recorded the medications administered (including paracetamol, antibiotics, hydroxychloroquine, remdesivir, azithromycin, Lopinavir/Ritonavir, and tocilizumab), as well as any oxygen therapy and ventilatory support. Data on the length of hospital stay as well as outcomes were also collected.

### Statistical Analysis

The participants' ages were stratified following the official age stratification used by the Statistics Centre Abu Dhabi (SCAD). The point prevalence was defined using the number of children in each age group diagnosed with Covid-19 during the study period as numerator, and the total number of children in that same age group living in the Al Ain district (latest SCAD census of 2016) as denominator. The 95% confidence intervals (ci) for the obtained age-specific point prevalence were calculated. Categorical variables were expressed as number and percentage. Normally distributed continuous variables (Wilk–Shapiro test) were expressed as mean ± standard deviation (SD), and non-normally distributed data were expressed as the median and interquartile range (IQR). All analyses were performed using the software STATA version 15.0 (STATA Corp., Texas).

## Results

### Epidemiology and Demographics

**Figure 1:**
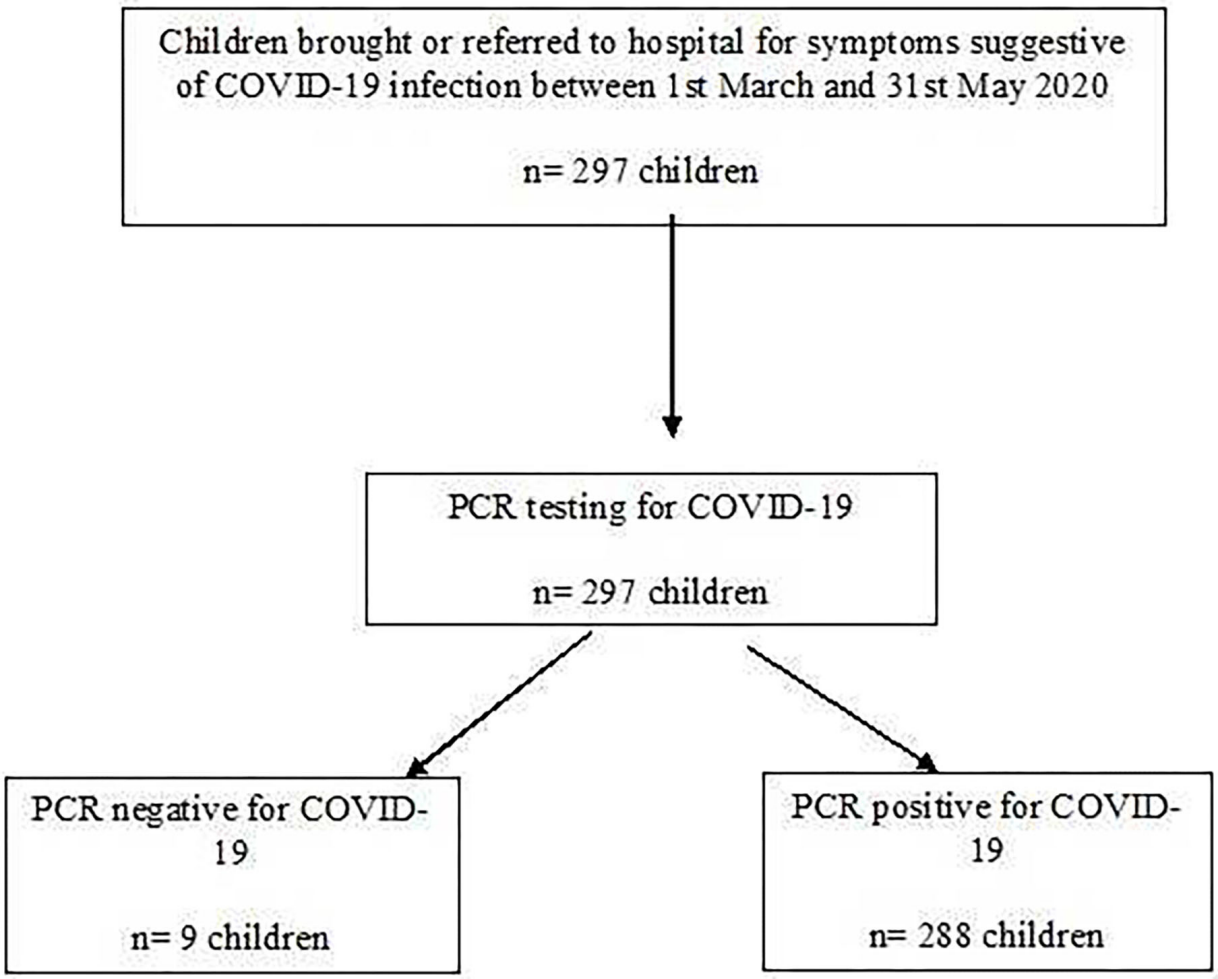
Flow chart for patient selection.

During the study period, 297 children were referred for assessment of whom 288 (96.9%) were confirmed to have SARS-CoV-2 infection (Flow chart Figure 1). Their mean age was 7.3 years (median 6.5, range 1 month to 16.9 years). The hospital admission was required for 193 (67%) children while 95 (33%) were discharged home from the emergency department.

**Figure 2:**
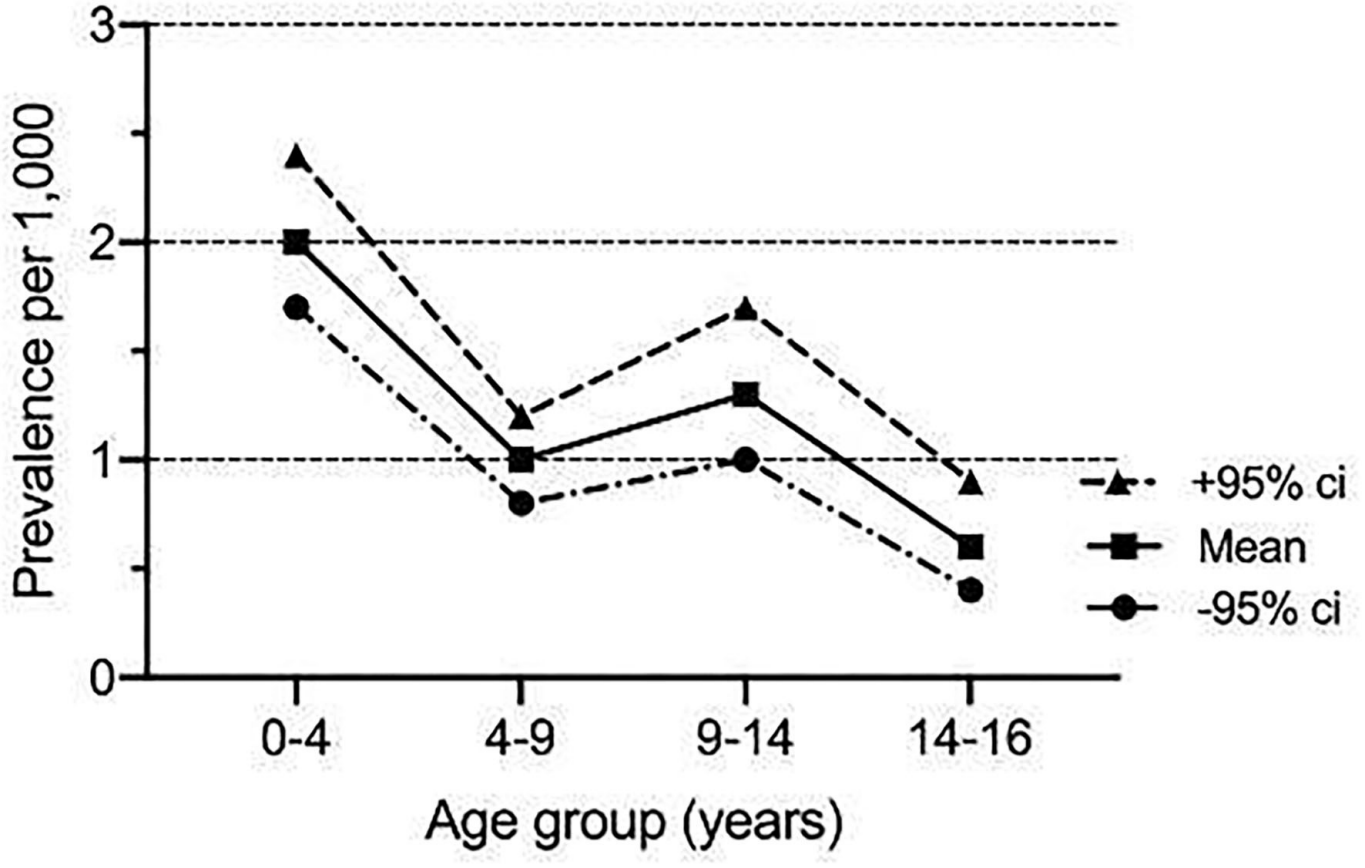
Age-specific point prevalence of COVID-19 infection in 288 children managed at Al Ain hospital between 1st March and 31st May 2020.

The age-specific point prevalence was the highest in children under 5 years of age (mean 2.0 per 1,000, 95% CI 1.7, 2.4) and decreased steadily to 0.6 per 1,000 (95% CI 0.4, 0.9) in those older than 14 years (Figure 2). This was also reflected in the age distribution, with 45% (*n* = 129) below the age of 5 years (Table 1). There was no significant difference in gender distribution, and the majority of children (*n* = 119, 40%) were UAE nationals (Table 1).

**Table 1:** Characteristics of 288 children managed with COVID-19 infection between 1st March and 31st May 2020.

**Age (years)**	6.5 (2.0, 12.3)
<5	129 (45)
5–9	58 (20)
9–14	66 (23)
14–16	35 (12)
**Sex**	
Male	148 (61.4)
Female	140 (48.6)
**Nationality**	
United Arab Emirates	119 (40)
India	52 (17)
Egypt	34 (12)
Others	83 (31)
**Children with ≥ 1 comorbidity**	27 (9.4)
Comorbidities^*^	56 (19.4)
Asthma	37 (13)
Seizures	7 (2.4)
Leukaemia	4 (1.4)
Cerebral palsy	3 (1.0)
Inborn error of metabolism	2 (0.7)
Down syndrome	2 (0.7)
Diabetes mellitus	1 (0.3)
Clinical features^*^	
Cough	130 (45)
Upper respiratory tract infection^**^	93 (32.3)
Sore throat	31 (10.8)
Lower respiratory tract infection	28 (9.7)
Temperature > 38^°^C	14 (4.8)
Headache	7 (2.4)
Anosmia	3 (1.0)
Abdominal pain	2 (0.7)
Vomiting	2 (0.7)
O_2_ saturation <92%	1 (0.3)
Diarrhoea	0 (0)
Acute respiratory distress syndrome	0 (0)
Neurological symptoms	0 (0)
Sepsis and septic shock	0 (0)
**Number of symptoms at presentation**	**Number of children percent**
None	76 (26.39)
1	99 (34.38)
2	76 (26.39)
3	32 (11.11)
4	4 (1.39)
5	1 (0.35)
Suspected exposure from^*^	
Family member	214 (74)
Unknown	67 (23.2)
Nanny	2 (0.7)
Hospital	2 (0.7)
Travel abroad	2 (0.7)
Friend	1 (0.3)

*Results expressed as the number of children (%)*.

* *Some children had one or more clinical features*.

** *Upper respiratory tract infection: common cold, sore throat, acute pharyngitis/tonsillitis, acute sinusitis, and acute otitis media*.

The contact exposure history revealed that the majority of children (*n* = 214, 74%) had been exposed to a family member with suspected or confirmed COVID-19. The source of exposure remained unknown in 67 (23.2%) children (Table 1).

### Comorbidities

Underlying health conditions were present in 56 children (19.4%), with asthma (*n* = 37, 13% of all children) being the most common (Table 1).

### Clinical Manifestations

Cough was the most common symptom (*n* = 130, 45%), followed by upper respiratory tract infection (*n* = 93, 32.3%) and sore throat (*n* = 31, 10.8%). Lower respiratory tract infection occurred in 28 children (9.7%) with 13/28 (46%) receiving antibiotics for secondary community-acquired pneumonia. Fever was present in 14 children (4.8%), anosmia in three (1.0%), and vomiting in two (0.7%). None of the children presented with acute respiratory distress syndrome, neurological symptoms, sepsis, or septic shock (Table 1). A total of 76 children (26.39) had no symptoms, the largest group of 99 children (34.38) had only one symptom, and 76 children (26.39) had two symptoms, whereas 32 (11.11) developed three symptoms, 4 children (1.39) had four symptoms, and only one child (0.35) developed five symptoms.

### Laboratory Findings

The results of the blood investigations, including white cell count, ferritin, lactate dehydrogenase, alanine transferase, aspartate aminotransferase, and international normalized ratio (INR) are shown in Table 2. Neutropenia (absolute neutrophil count or ANC < 1.5 × 10^9^/L) was observed in 10.4% and thrombocytopenia (<150 platelets × 10^9^/L) in 72% of children, with varying degrees of severity as detailed in Supplement 2.

**Table 2:** Investigations results of 288 children managed with COVID-19 infection between 1st March and 31st May 2020.

	***n* (%)**
**Laboratory findings**	
Total white blood cell count (x10^9^/L)	7.4 [5.4, 9.6]
Hemoglobin concentration (g/L)	125 [116, 133]
Platelet count (x10^9^/L)	87 [255, 372]
platelet count <150 x 10^9^/L	208 [72.2]
Neutrophils count (x10^9^/L)	2.3 [1.6, 3.6]
Ferritin (ng/mL)	57 [37, 85]
Lactic dehydrogenase (U/L)	245 [208, 290]
Alanine aminotransferase (U/L)	16 [12, 21]
Aspartate aminotransferase (U/L)	28 [21, 34]
International normalized ratio (INR)	1.07 ± 0.1
Prothrombin time (seconds)	11.5 ± 0.7
**Imaging**	
Chest X-ray done to all 288/288 (100) Chest X-ray opacities	19 (9)
Right-sided	7 (50)
Left-sided	1 (7)
Bilateral	6 (43)
Abnormal computed tomography	1 (6)
**Microbiology**	
Respiratory COVID-19 PCR positive	288 (100)
**Respiratory co-infections**	9 (3.1)
Human rhinovirus	3 (1.0)
Streptococcus A	3 (1.0)
Adenovirus	1 (0.3)
*Streptococcus pneumonia*	1 (0.3)
*Haemophilus influenzae*	1 (0.3)
**Pharmacological treatment**	
Hydroxychloroquine	17 (5.9)
Other antibiotics	48 (16.6)
Azithromycin	2 (0.7)
Lopinavir/Ritonavir	0 (0)
Tocilizumab	0 (0)
**Respiratory support**	
Invasive respiratory support	0 (0)
Non-invasive respiratory support (high flow)	4 (1.4)
**Length of hospital stay (days)**	3.3
**Death**	0 (0)

*Results expressed as the number of children (%), or mean value ± SD, or median [iqr]*.

All patients were also screened for other respiratory pathogens (Table 2): coinfection occurred in nine patients (3%): three were coinfected with human rhinovirus, another three with *Group A Streptococcus*, and one child each with adenovirus, *Streptococcus pneumoniae*, or *Haemophilus influenzae*.

### Radiological Findings

Nineteen patients (9%) had abnormal imaging studies (chest X-ray and chest computed tomography), with bilateral changes in six (43%), right-sided lung involvement in seven (50%), and only one child (7%) having left-sided lung involvement (Table 2).

### Treatment and Outcome

Most patients had a mild course requiring only supportive treatment with antipyretics. Hydroxychloroquine was used in 17 (5.9%) children, including two who also had azithromycin. Antibiotics were administered to 48 (16.6%). None of the children required invasive respiratory support (ventilation through an endotracheal tube or tracheostomy), but four patients (1.4%) required non-invasive respiratory support (ventilation of the upper airways precluding endotracheal tube or tracheostomy). The median length of hospital stay was 3.3 days [1.9, 5.9]. There were no deaths in hospital including children with comorbidities (Table 2).

## Discussion

We have established that, throughout childhood, the prevalence of COVID-19 decreases with age, in contrast to studies from China (18, 19). The reasons for this difference are unclear, and, although the underlying genetic difference in susceptibility to SARS-CoV-2 between the two populations may be hypothesized, this remains entirely speculative. Nevertheless, our results confirm that children very often have no or mild disease (18, 19).

A history of sick contact was found in only 75% of our patients, a lower proportion than previously reported (18). We admit to the possibility of recall bias and that the documentation of contact was not always complete. The common signs or symptoms in our cohort are largely similar to previous reports, including fever, cough, and rhinitis (20). Respiratory symptoms associated with lower respiratory tract infection were less common (9.7%) than in previous reports (9–17), the reasons for which are not immediately obvious.

As asthma is one of the most common chronic diseases in childhood, it is not surprising that it was also the most frequent comorbidity in our cohort. However, it has already been observed that asthma does not increase the susceptibility of affected patients to SARS-CoV2, nor does it increase the morbidity in COVID-19 (21). In this cohort of patients, only two of the 37 children with asthma developed a mild exacerbation of their symptoms requiring treatment with inhaled bronchodilators, in addition to their regular inhaled corticosteroids.

We reviewed all nine primary publications that had reported ≥ 50 children with COVID-19 and compared their findings with ours (Supplement 3) (9–17). Our study has the 2nd largest number of affected children (*n* = 288) after one report on 582 children from 25 European countries (11). Common findings included a slight predominance of boys and a broadly similar proportion of preexisting comorbidities. Unlike other reports (5, 22–26), a minority of our patients presented with abdominal pain or vomiting, while none had neurological symptoms, sepsis, or septic shock. Acute respiratory distress syndrome (ARDS) in COVID-19 patients, previously reported to range from 3 to 5.8% (22, 23), and 17 to 42% in those with established COVID-19 pneumonia (20, 24), was not observed in our patients, including the 10% who had COVID-19 pneumonia. The reasons for the observed difference remain unclear, and we can only speculate possible genetic variations behind these differences. Confirming similar studies reporting a low rate of intensive care admission (22), none of our patients required critical care management. Although the presence of comorbidities previously reported to be a risk factor for critical illness with COVID-19 in children (19), this was not confirmed in our study. Similarly, we did not confirm any previously described association between social factors, ethnicity, and the increased risk of prolonged hospital stay and intensive care admission (23, 25). Neither have we observed the recently described pediatric inflammatory multisystem syndrome (PIMS) with multiorgan involvement that shares some features with Kawasaki disease and toxic shock syndrome (5, 26–30). A possible reason is that, as PIMS usually develops after a time interval following infection with SARS-CoV-2, it might not have been observed during the children's short hospital stay. However, until the time of writing this paper, and to the best of our knowledge, telemedicine or face-to-face clinic follow-up has not revealed that any of our patients have suffered from this complication after discharge from the hospital, nor those referred to isolation facilities.

The laboratory findings are also in contrast with recently published reports. While we observed no lymphopenia, in contrast to its prevalence of 16% in a previous report (14), we witnessed a hitherto unreported high prevalence of neutropenia and thrombocytopenia, not described in earlier studies (14, 16, 17).

The pharmacological interventions that we used were not evidence-based and were probably not any better than supportive treatment (30). The hospital course was uncomplicated in most patients, with a median hospital stay of 3.3 days, associated with a favorable outcome and no mortality. The shorter hospital stay we described, in comparison with previous reports (Supplement 3), probably reflects the benign course as none of the affected children developed serious pulmonary or systemic complications (9–17). The reason for the marginal difference of this study with no severe morbidity nor mortality reported is not clear but may be multifactorial involving, alone or in combination, genetic, immunological, epidemiological, environmental, and economic factors. As adults with COVID-19 managed in our hospital developed the full range of complications described in this condition, with high morbidity and mortality, we believe that it is very unlikely that a different strain of SARS-Cov2 in our city could explain the milder course in our pediatric population. Further studies which could involve genotyping the virus as well as measuring viral loads as it correlates with the severe manifestations of the disease may be required to clarify any role of the speculated factors in the benign course that we observed in affected children.

A strength of the study is that our cohort of 288 children with COVID-19 represents one of the largest single-center studies so far. However, unlike other reports, it is truly representative of the child population in Al Ain city because all affected children in the city were managed in the single designated referral hospital for COVID-19. The established age-specific prevalence of that infection is therefore valid and truly representative of children in our city.

Limitations to the study include its retrospective nature, restricted therefore by data already collected. In addition, we had no systematic follow-up data on the children after their discharge from hospital to measure the time taken for the virus to disappear from their nasopharynx or to assess their subsequent seropositivity to SARS-CoV-2, or study the effect of the associated factors on long-term outcomes. Furthermore, as it was from a single center in Al Ain city, our results may not reflect the entire spectrum of COVID-19 disease in children throughout the country in contrast to other studies (5, 26–29). Another limitation is the lack of statistical comparison between the selected population in terms of age, gender, and contact history. We acknowledge the possibility of incomplete recruitment if some asymptomatic children, with no contact history, were not referred to our hospital and were therefore not included in our cohort.

## Conclusions

Children with COVID-19, in contrast to adults, are often asymptomatic or demonstrate mild illness, and in our cohort even those with comorbidities did not suffer any acute respiratory syndrome or other major complications of SARS-COV2. Our report demonstrates an even milder disease than already published, with no report of severe morbidity or mortality. Added to the calculated age-specific prevalence of that infection in children, our results will help in planning health care services if future resurgences of COVID-19 occur. Future studies mitigating against the limitations of the study are needed. Our findings should also reassure the parents and caregivers of children with comorbidities that they do not seem to have a higher COVID-19 illness severity than other healthy children.

## Data Availability Statement

The raw data supporting the conclusions of this article will be made available by the authors, without undue reservation.

## Ethics Statement

The studies involving human participants were reviewed and approved by the Ethics Committee, The Department of Health, Abu Dhabi Health Authority, United Arab Emirates. Informed consent to participate in this study was waived as it was a retrospective study with all collected data being anonymised.

## Author Contributions

All authors were involved in the clinical care of the patients and data collection. HN performed the data analysis. All authors contributed to drafting and critically revising the manuscript, intellectual content, and approved its final version.

## Conflict of Interest

The authors declare that the research was conducted in the absence of any commercial or financial relationships that could be construed as a potential conflict of interest.
