# Supplementary material for: Covid-19 in Children and Young Adolescents in Al Ain, United Arab Emirates- a Retrospective Cross-Sectional Study
Source: Front Pediatr. 2021 Jan 18;8:603741. doi: 10.3389/fped.2020.603741 (PMC7848192; doi:10.3389/fped.2020.603741)
Supplement: Supplementary file 1 [file Data_Sheet_1.pdf]

SUPPLEMENTS

Supplement 1 (S1)

Nasopharyngeal sample collection

We used the Cobas® (Roche Rotkreuz, Switzerland) flocked swabs for that purpose. During the sample collection, a non–touch technique was used by holding the flocked swab with fingers placed on the scoreline, and the patient seated with head tilted to the back 70 degrees. The flocked swab was gently inserted into the nostril along the nasal septum floor and pushed straight back until the posterior nasopharynx was reached (distance from nostrils to the external opening of the ear). This was followed by rotating the swab several times, while the swab remained in contact with the nasopharyngeal wall, for a minimum of five seconds. Finally, the flocked swab was immediately placed into 3 mL of BD Universal Viral Transport (UVT) medium and broken (snaped) off swab at the indicator line, before replacing the cap and screw on securely[8,9&10]. Samples were kept at a temperature between 2-8 degrees Celsius until processed.

Real-time polymerase chain reaction (RTPCR) testing of the collected specimens.

We used the Allplex™ 2019-nCoV Assay Seegene PCR kit (Seegene Inc., Seoul, Republic of Korea). A positive COVID-19 result was defined by the detection of a three-gene (E, RdRP, and N gene). If only the E gene was detected, the test was repeated using different assays (Cobas® SARS-CoV-2, Roche, Rotkreuz, Switzerland) and then reported as positive if that test was positive. If only the E gene was detected that second time, the test was reported as "presumptive positive." Moreover, all samples with cycle threshold (Ct) value >35 (indicating a low viral load) were retested on the Cobas® SARS-CoV-2 platform.

Supplement 2 (S2) Degree of neutropenia, defined by the absolute neutrophil count (ANC), and of thrombocytopenia in 288 children with Covid-19 infection

**ANC x 10^9/L**	**n**	**%**	**Cumulative %**
Profound (ANC < 0.1)	2	0.69	0.69
Severe (ANC 0.1- 0.5)	5	1.74	2.43
Moderate (ANC 0.5- 1.0)	14	4.86	7.29
Mild (ANC 1.0- 1.5)	30	10.42	17.71
Normal (ANC > 1.5)	187	64.93	82.64
Missing data	50	17.36	100.00
**Platelet count x 10^9/L**			
> 150	80	27.78	27.78
75- 150	105	36.46	64.24
50-75	45	15.62	79.86
25-50	32	11.11	90.97
< 25	26	9.03	100.00
Total	288	100.00	

Supplement Table (S3)

Comparison with other primary reports of ≥ 50 children with confirmed COVID-19 infection (except (17) as it is the only other available report from the Gulf countries). Meta-analyses, systematic reviews and studies of children from intensive care units were excluded.

	**Our study**	**Germany** (9)	**USA** (10)	**Europe** (11)	**UK** (12)	**Turkey** (13)	**China** (14)	**Italy** (15)	**China** (16)	**Saudi Arabia** (17)
Number of children	288	128	67	582	65	81	50	100	74	24
Period	1 March to 31 May 2020	18 March to 4 May 2020	15 March to 13 April	1 to 24 April 2020	1 March to 15 May 2020	5 March to 5 May 2020	21 January to 14 February	3 to 27 March 2020	20 Jan to 27 February 2020	1 March to 13 May 2020
Number of institutions	1	66	1	82	1	1	1	17	1	1
Age in years as mean ± SD, or median [iqr]	6.5 [2.0, 12.3]	--	13.1 [0.4, 19.3]	5 [0.5, 12]	9 [0.9, 14]	9.5 [3.1, 15.1]	2.5 (0.9, 7.0)	3.3 [range 0, 17.5]]	6.0 [range 0.1, 15.1)	8.4 ± 5.8
Age (years)										
<5	129 (45)	84 (66)	--	291 (51)	--	33 (41)	--	55 (55)	--	8 (33)
5-9	58 (20)	18 (14)	--	94 (16)	--	17 (21)	--	21 (21)	--	7 (29)
10-14	66 (23)	14 (11)	--	196 (34)	--	48 (59)	--	24 (24)	--	9 (38)
15-18	35 (12)	12 (9)	--		--		--		--	
Males	148 (61.4)	64 (50)	31 (67.4)	311 (53)	--	44 (54)	28(56)	57 (57)	44 (59.5)	13 (54)
Comorbidity	56 (19.4)	--	--	145 (25)	--	14 (17)	15 (30)	27 (27)		--
Co-infection	9 (3.1)	6 (4.7)	--	29 (5)	--	6 (14)	--	--	--	--
Gastrointestinal symptoms	4 (1.4)	22 (17)	0 (0)	128 (22)	--	3 (4)	3 (6)	19 (19)	3 (4.1)	--
Upper respiratory tract infection	93 (32.3)	--	--	313 (54)	--	4 (9)	8 (16)	22 (22)	24 (32.4)	8 (33)
Lower respiratory tract infection	28 (9.7)	--	--	143 (25)	--	33 (75)	--	28 (28)	29 (39.2)	12(50)
Acute respiratory distress syndrome	0 (0)	2(2)	10 (15)	--	--	--	--	9 (9)	--	--
Neurological symptoms	7 (2.4)	2(2)	0 (0)	--	--	0 (0)	--	11 (11)	0 (0)	--
Sepsis and septic shock	0 (0)	7(6)	7 (10)	19 (3)	--	--	--		--	--
Intensive care	0 (0)	16 (12.5)	13 (28)	48 (8)	29 (45)	2 (5)	--	9 (9)	--	--
Invasive ventilation	0 (0)	7 (5)	6 (10)	25 (4)	18 (27)	--	--	9 (9)	0 (0)	--
Median length of hospital stay (range) or [iqr] in days or mean ± SD	3.3 [1.9, 5.9].	3 (1-26)	3 [2, 4]	--	--	5 [4, 10]	11 (8.2, 13.8)	--	--	5.6 ± 4.0
Death	0 (0)	1 (0.8)	1 (1.5)	--	9 (14)	9 (11)	0 (0)	0 (0)	--	0 (0)
